# PGPB Bacillus Megaterium AFI1 and Paenibacillus Nicotianae AFI2 Improve Nutrient Uptake and Stimulate Adaptation of Wheat Under Nickel Exposure

**DOI:** 10.3390/ijms27115041

**Published:** 2026-06-02

**Authors:** Veronika N. Pishchik, Galina V. Mirskaya, Polina S. Filippova, Vitaliy E. Vertebny, Victoria I. Dubovitskaya, Dmitriy V. Kudryavtcev, Olga A. Bortsova, Yuriy V. Khomyakov, Pavel Y. Kononchuk, Vladimir K. Chebotar

**Affiliations:** 1 All-Russia Research Institute for Agricultural Microbiology, Podbelskogo hwy, 3, Pushkin, St. Petersburg 196608, Russia; dv.kudryavtsev@arriam.ru (D.V.K.); bortsova17@yandex.ru (O.A.B.); 2 Agrophysical Scientific Research Institute, Grazhdansky pr. 14, St. Petersburg 195220, Russia; galinanm@gmail.com (G.V.M.); verteb22@mail.ru (V.E.V.); vikot85@mail.ru (V.I.D.); himlabafi@yandex.ru (Y.V.K.); 79117717774@yandex.ru (P.Y.K.); 3 St. Petersburg Federal Research Center of the Russian Academy of Sciences, North-West Center of Interdisciplinary Researches of Problems of Food Maintenance, Podbelskogo hwy, 7, Pushkin, St. Petersburg 196608, Russia; tipolis@yandex.ru

**Keywords:** nickel stress, PGPB, *Bacillus megaterium* AFI1, *Paenibacillus nicotianae* AFI2, wheat, mineral composition of wheat, antioxidant protection

## Abstract

Due to the increased anthropogenic load, crops are polluted with heavy metals, including nickel (Ni). This is a serious environmental problem, as Ni penetrates barrier-free into cereal crops and accumulates in the grains used by humans and animals for food. Wheat is one of the main staple crops, cultivated in many countries. This study suggested that plant growth promoting bacteria (PGPB) with varying enzymatic activities could help wheat plants to cope with Ni stress by reducing Ni toxicity and regulating the metal’s homeostasis. PGPB *Bacillus megaterium* AFI1 has a strong phosphate-solubilizing activity and produces siderophores, while *Paenibacillus nicotianae* AFI2 has nitrogen-fixing and silicate-solubilizing activities. Both strains produce indole and polysaccharides and have 1-aminocyclopropane-1-carboxylate (ACC) deaminase activity. PGPB under Ni exposure (100 mg/kg of soil) significantly increased grain yield (by 34–42%) and decreased (by 20–33%) Ni content in wheat grains. PGPB also decreased malondialdehyde (MDA) and H_2_O_2_ levels in wheat plants under Ni stress. The contents of iron (Fe), boron (B), nitrogen (N) and phosphorus (P) decreased significantly and potassium (K) and zinc (Zn) oppositely increased significantly in all plant organs under Ni exposure. The inoculation with AFI1 mainly increased P and Fe, and the inoculation with AFI2 increased N and silica (Si) in wheat grains under Ni stress. In our experiments, under nickel exposure PGPB *Bacillus megaterium* AFI1 and *Paenibacillus nicotianae* AFI2 increased antioxidant protection of plants by decreasing the level of stress ethylene and regulating the homeostasis of nutrients in wheat plants. These PGPB can be considered as promising candidates for the development of biologicals to be used for growing plants in soils with low levels of nickel contamination.

## 1. Introduction

Contamination of agricultural soils with heavy metals (HMs) poses a serious threat to food security and human health [1,2]. High concentrations of HMs in the soil contribute to the contamination of agricultural land [1]. It has a negative effect on the assimilation of elements from the soil by plants, inhibits plant growth and causes toxic symptoms, including wilting of plants [3,4,5]. Nickel (Ni) in high concentrations (20–60 mg/L) is a toxic element for agricultural plants and, due to barrier-free penetration, is able to accumulate in the generative organs of plants [6,7,8]. Ni in high concentrations causes ion imbalance and changes the mineral composition of plants, and also inhibits enzyme activity, disrupts mitochondrial function and provokes oxidative stress [9]. This leads to numerous metabolic disorders in the plants [10,11]. Ni interaction with functional groups of proteins leads to disruption of their functional activity [12].

PGPB have been successfully used in agriculture in many countries to increase crop yields and protect plants from various abiotic stresses [13], including as a method for reducing the toxicity of contaminated soils [14]. PGPB have the ability to bind toxic metals through mechanisms such as biosorption, bioaccumulation, and transformation, which reduce the negative impacts of HMs [15,16,17]. The functional groups of bacterial proteins bind HM ions. The interaction of HM ions with hydroxyl, carbonyl, and amino groups and with negatively charged phosphate and carboxyl groups of proteins was recently revealed using infrared analysis [12]. Reduction in HM toxicity to plants through the influence of microorganisms can be achieved by reducing metal uptake by plants [18,19], as well as by removing HM from nutrient media [20]. PGPB of the genus *Bacillus* are known to be resistant to high concentrations of Ni and can grow on a medium with a Ni concentration of 80 mg/L [21] and may be used in agriculture as bioinoculants for alleviating abiotic stresses [22]. For instance, recently a strain from the genus *Paenibacillus* has been described as a potential multi-stress-tolerant microbial inoculant [23].

It is known that PGPB, through various mechanisms of action, are capable of improving plant growth and quality under Ni stress. These mechanisms include phosphate-mobilizing and nitrogen-fixing activities, production of phytohormones, siderophores, ribonucleases, lipopeptides, polysaccharides and enzymes [24,25,26,27]. There is also evidence of enhanced mineral nutrition and an increase in the crude ash content under the influence of the inoculation with PGPB [28]. The improvement of mineral nutrition is achieved through bacterial enzymes and organic acids, which leads to the breakdown of rhizosphere substances through bacterial metabolism [29].

The complex system of interactions between PGPB and plants represents a set of factors through which PGPB positively influence plant growth and development. PGPB and bacterial associations have similar effects on growth performance, despite the fact that bacteria produce different metabolites and have different characteristics [24,30].

Wheat is one of the main cereal crops and its cultivation ensures food security worldwide [31]. The use of biologicals in growing wheat in various climatic conditions and stresses is relevant for agriculture in many countries. Some experiments have been conducted on the effects of PGPB on wheat plants under Ni stress at minimal toxic concentrations [11,32] and high concentrations of nickel ions [28,33].

It has been shown in field experiments that bacteria of the genus *Azospirillum* help wheat plants to cope with mild Ni stress (4 mg/kg of soil) [32]. PGPB *Bacillus megaterium* AFI1 and *Paenibacillus nicotianae* AFI2 stimulated wheat plants growing at minimal toxic concentrations of Ni ions under hydroponic conditions [11]. It was previously shown that the strain *Bacillus subtilis* BM2 decreased the Ni concentration in wheat grains by 6.2% when growing winter wheat in the presence of 870 mg/kg Ni in the soil [33]. However, the strains *Pseudomonas fluorescens* 20 and 21 and *Pseudomonas putida* 23 oppositely increased Ni content in wheat grains [28].

We hypothesized that PGPB strains *Bacillus megaterium* AFI1 and *Paenibacillus nicotianae* AFI2 could change Ni homeostasis in wheat plants under severe Ni stress (100 mg/kg of soil) and help wheat plants to overcome Ni stress. To confirm this hypothesis, we studied the effect of PGPB under Ni exposure on the wheat growth, antioxidant status, trace elements and macronutrients uptake and wheat organ nutrient distribution in vegetative experiments under controlled conditions. This paper highlights the PGPB influence on Ni homeostasis and nutrient uptake by wheat plants under severe Ni stress.

## 2. Results

### 2.1. Properties of Bacteria

The PGPB strains AFI1 and AFI2 have ACC deaminase, amylolytic, proteolytic, and phosphate-mobilizing activities, and produce polysaccharides and indoles (Table 1).

**Figure 1:**
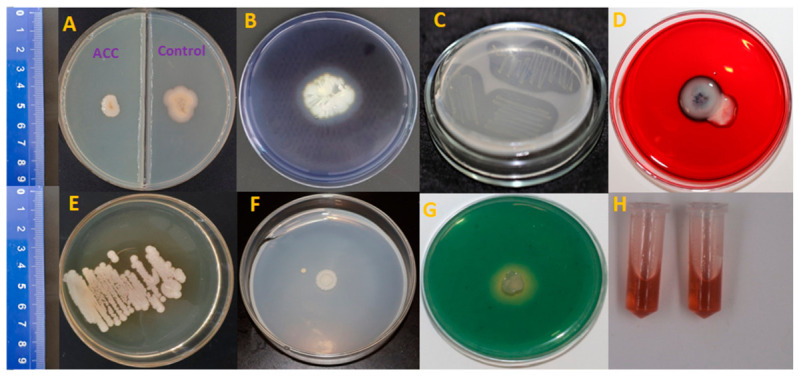
Metabolic activity of *Bacillus megaterium* AFI1 bacterium. Notes: (**A**) ACC activity, (**B**) amylolytic activity, (**C**) phosphate-mobilizing activity, (**D**) exopolysaccharides activity, (**E**) growth on the LB medium with 100 mg/L Ni^2+^, (**F**) silicate-solubilizing activity, (**G**) siderophore-producing activity, (**H**) indole-producing activity.

**Figure 2:**
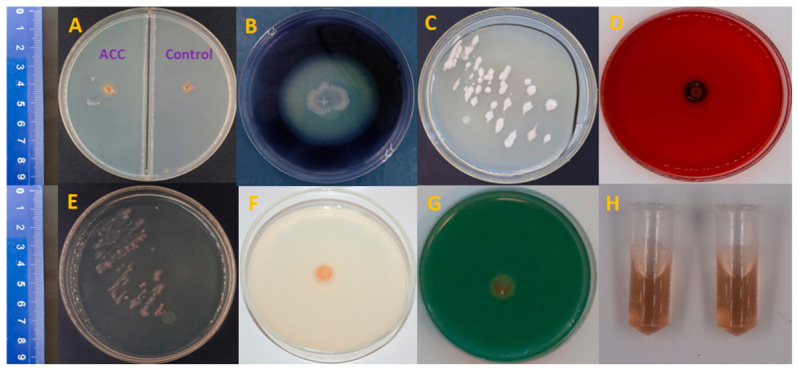
Metabolic activity of *Paenibacillus nicotianae* AFI2 bacterium. Notes: (**A**) ACC activity, (**B**) amylolytic activity, (**C**) phosphate-mobilizing activity, (**D**) exopolysaccharides activity, (**E**) growth on the LB medium with 100 mg/L Ni^2+^, (**F**) silicon-mobilizing activity, (**G**) siderophore-producing activity, (**H**) indole-producing activity.

Both strains were grown on LB medium, containing 100 mg/L Ni^2+^ (Figure 1 and Figure 2). The strain AFI1 produced siderophores (Figure 1) and possessed strong phosphate-solubilizing activity, and the strain AFI2 has nitrogen-fixing and silicate-solubilizing activity (Figure 2).

When studying the resistance of bacteria to different concentrations of Ni ions, it was found that both strains were able to grow on a liquid LB medium at the Ni concentration of 50 and 100 mg/L g/L (Figure S1).

The strain AFI1 absorbed 22% and 10% of Ni ions from the supernatant when grown on a medium with a concentration of 50 and 100 mg/L accordingly. The strain AFI2 absorbed only 10 and 2% Ni ions from the supernatant when grown on LB medium with similar concentrations.

### 2.2. The Effect of Ni and Bacteria on the Activity of Antioxidant Enzymes and the Content of MDA, Photosynthetic Pigments and Parameters of the Wheat Flag Leaf

The positive effect of PGPB on wheat plants under Ni exposure was expressed in the regulation of the homeostasis of trace elements and macronutrients, as well as in the increase in antioxidant protection. PGPB decreased malondialdehyde (MDA), H_2_O_2_ levels and antioxidant enzyme activities in the leaves of wheat plants at the shooting stage (Table 2). PGPB also increased the contents of chlorophylls (chls) and carotenoids (car) (Table 3). PGPB significantly decreased (1.3–1.7-fold) Ni content in wheat leaves under Ni exposure.

The effect of Ni stress was expressed in an increase in the activity of antioxidant enzymes: peroxidase (POX) and catalase (CAT) by 32 and 27% respectively (Table 2). The levels of MDA and H_2_O_2_ were increased by 31% and 60% respectively. The positive effect of the inoculation with PGPB was pronounced in a decrease in the level of MDA by 18–21%, and H_2_O_2_ by 47–54%. The activities of POX and CAT were also decreased by 7–41% and 4–7% respectively.

The contents of photosynthetic pigments in leaves were decreased under Ni exposure—by 21% (chl *a*), by 33% (chl *b*), and by 5% (car) (Table 3). PGPB reduced the toxic effect of Ni and increased the contents of chlorophylls, chl *a* by 37–53%, chl *b* by 39–52%, and car by 32–54%, depending on the inoculation options. Strain AFI2 as well as both strains together maximized the contents of chl and car.

The inoculation with PGPB significantly increased the growth parameters (length and width) of the wheat flag leaf under both control and Ni stress conditions, increasing the leaf surface area by 25–37% and by 15–24%, respectively (Table S1). Moreover, under the Ni exposure strain AFI1 maximum increased the flag leaf surface area.

### 2.3. Effect of PGPB on Wheat Plant Biomass and Ni Accumulation by Wheat Plants

PGPB AFI1 and AFI2 increased grain biomass by 25% and 45%, respectively, compared with the control (Table 4). Plant growth was decreased under Ni exposure. Grain yield was significantly reduced (by 34%) under Ni exposure. The inoculation with AFI1 and AFI2 strains under Ni exposure improved the biomass of plant roots. Strain AFI1 improved root biomass by 33%, and co-inoculation with both strains increased root biomass by 41%. Strain AFI2 greatly increased the biomass of the aboveground part of plants by 30% under Ni stress. Strain AFI1 greatly increased by 42% grain yield, and co-inoculation with both strains AFI1 and AFI2 increased by 47% grain yield under Ni exposure (Table 4).

**Figure 3:**
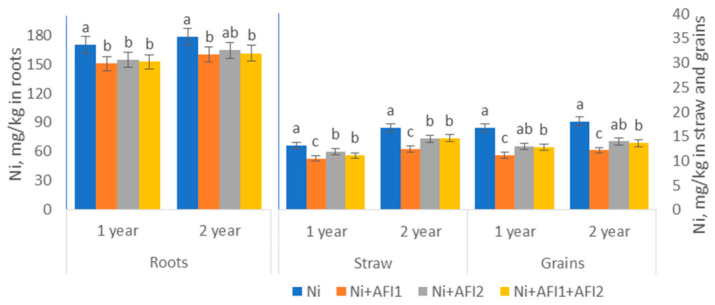
The effect of inoculation with AFI1 and AFI2 strains on Ni accumulation in various wheat organs under Ni stress. Notes: Ni: Non-inoculated wheat plants, growing under Ni stress (100 mg Ni^2+^ per 1 kg of soil). AFI1 and AFI2: Wheat plants inoculated with appropriate strains *Bacillus megaterium* AFI1 and *Paenibacillus nicotianae* AFI2. Bars with different letters are significantly different at *p* ≤ 0.05, as determined by Duncan’s multiple range test. Wheat plants cv. Leningradskaya 6 were grown under controlled conditions during two vegetation experiments (80 days).

A study of Ni distribution in various parts of the wheat plant revealed that the Ni was accumulated mostly in the roots (162.6 mg/kg) after wheat ripening. Ni contents in the grains (18.3 mg/kg) and in the straw (14.4 mg/kg) were 11 and 9 times lower than those in the roots, respectively, on average over two years (Figure 3).

The average coefficient of Ni bioaccumulation CBA in wheat grain (Table S2) was higher than in the straw (Table S3) and was significantly reduced by bacterial inoculation. Ni CBA in wheat grains was decreased by the strain AFI1 1.5-fold, and by the strain AFI2 1.3-fold. The inoculation with PGPB also resulted in a significant 1.2–1.3-fold decrease in the average coefficient of translacation (CT) in wheat grains (Table S2).

### 2.4. Effect of Ni Stress and Bacterial Inoculation on Trace Element and Macronutrient Contents in Various Wheat Organs

**Figure 4:**
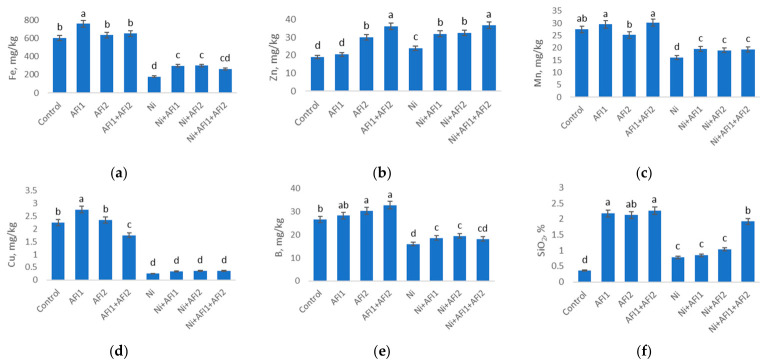
The effect of Ni exposure and inoculation with PGPB on the content of trace elements in wheat roots (in the ripeness stage). (**a**) Fe; (**b**) Zn; (**c**) Mn; (**d**) Cu; (**e**) B; (**f**) SiO_2_. Notes: Ni: Non-inoculated wheat plants, growing under Ni stress (100 mg Ni^2+^ per 1 kg of soil). AFI1 and AFI2: Wheat plants inoculated with appropriate strains *Bacillus megaterium* AFI1 and *Paenibacillus nicotianae* AFI2. Bars with different letters are significantly different at *p* ≤ 0.05, as determined by Duncan’s multiple range test. Wheat plants cv. Leningradskaya 6 were grown under controlled conditions during two vegetation experiments (80 days).

**Figure 5:**
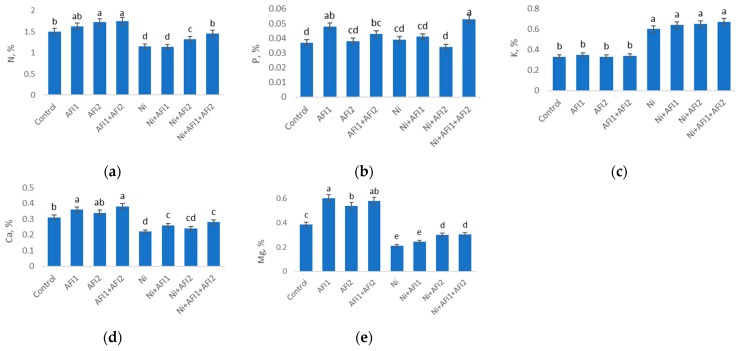
The effect of Ni exposure and inoculation with PGPB on the content of macronutrients in wheat roots (in the ripeness stage): (**a**) N; (**b**) P; (**c**) K; (**d**) Ca; (**e**) Mg. Notes: Ni: Non-inoculated wheat plants, growing under Ni stress (100 mg Ni^2+^ per 1 kg of soil). AFI1 and AFI2: Wheat plants inoculated with appropriate strains *Bacillus megaterium* AFI1 and *Paenibacillus nicotianae* AFI2. Bars with different letters are significantly different at *p* ≤ 0.05, as determined by Duncan’s multiple range test. Wheat plants cv. Leningradskaya 6 were grown under controlled conditions during two vegetation experiments (80 days).

**Figure 6:**
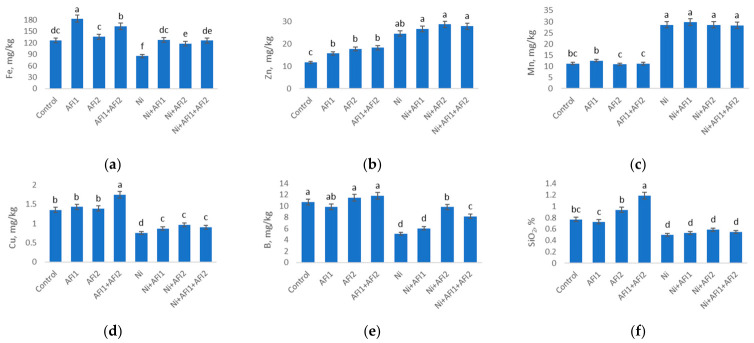
The effect of Ni exposure and the inoculation with PGPB on the content of trace elements in wheat straw (in the ripeness phase): (**a**) (Fe); (**b**) Zn; (**c**) Mn; (**d**) Cu; (**e**) B; (**f**) SiO_2_. Notes: Ni: Non-inoculated wheat plants, growing under Ni stress (100 mg Ni^2+^ per 1 kg of soil). AFI1 and AFI2: Wheat plants inoculated with appropriate strains *Bacillus megaterium* AFI1 and *Paenibacillus nicotianae* AFI2. Bars with different letters are significantly different at *p* ≤ 0.05, as determined by Duncan’s multiple range test.

**Figure 7:**
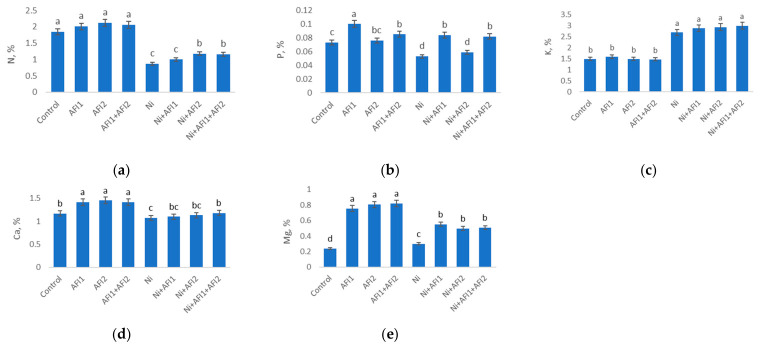
The effect of Ni exposure and inoculation with PGPB on the content of macronutrients in wheat straw (in the ripeness stage)**:** (**a**) N; (**b**) P; (**c**) K; (**d**) Ca; (**e**) Mg. Notes: Ni: Non-inoculated wheat plants, growing under Ni stress (100 mg Ni^2+^ per 1 kg of soil). AFI1 and AFI2: Wheat plants inoculated with appropriate strains *Bacillus megaterium* AFI1 and *Paenibacillus nicotianae* AFI2. Bars with different letters are significantly different at *p* ≤ 0.05, as determined by Duncan’s multiple range test. Wheat plants cv. Leningradskaya 6 were grown under controlled conditions during two vegetation experiments (80 days).

**Figure 8:**
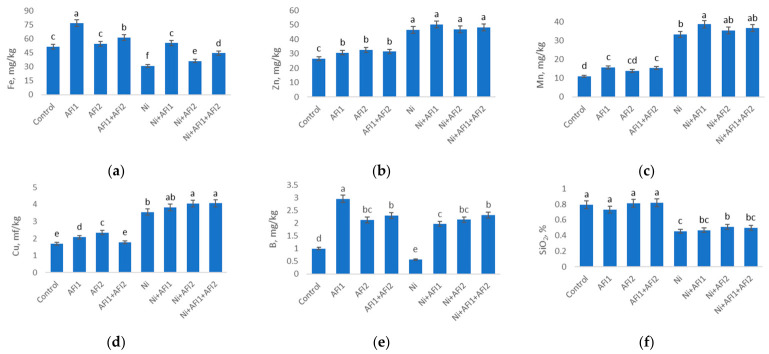
The effect of Ni exposure and the inoculation with PGPB on the content of trace elements in wheat grain (in the full ripeness phase): (**a**) (Fe); (**b**) Zn; (**c**) Mn; (**d**) Cu; (**e**) SiO_2_; (**f**) B. Notes: Ni: Non-inoculated wheat plants, growing under Ni stress (100 mg Ni^2+^ per 1 kg of soil). AFI1 and AFI2: Wheat plants inoculated with appropriate strains *Bacillus megaterium* AFI1 and *Paenibacillus nicotianae* AFI2. Bars with different letters are significantly different at *p* ≤ 0.05, as determined by Duncan’s multiple range test.

**Figure 9:**
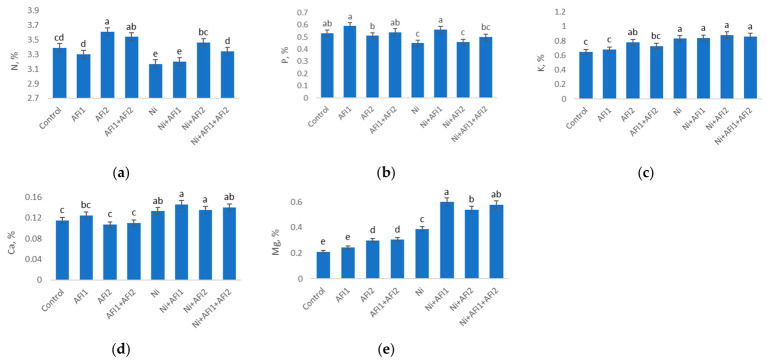
The effect of Ni exposure and inoculation with PGPB on the contents of macronutrients in wheat grain (in the full ripeness phase): (**a**) N; (**b**) P; (**c**) K; (**d**) Ca; (**e**) Mg. Notes: Ni: Non-inoculated wheat plants, growing under Ni stress (100 mg Ni^2+^ per 1 kg of soil). AFI1 and AFI2: Wheat plants inoculated with appropriate strains *Bacillus megaterium* AFI1 and *Paenibacillus nicotianae* AFI2. Bars with different letters are significantly different at *p* ≤ 0.05, as determined by Duncan’s multiple range test.

The addition of Ni to the soil caused changes in trace element and macronutrient contents in the straw, grains, and roots of wheat plants (Figure 4, Figure 5, Figure 6, Figure 7, Figure 8 and Figure 9). Specifically, maximum absorption of zinc (Zn) by roots was noted under Ni exposure (Figure 4b). The greatest impact of the Ni toxic dose was a significant decrease in copper (Cu), magnesium (Mg), and iron (Fe) contents in plant roots (Figure 4).

Mg and Fe contents were significantly reduced in wheat roots under Ni stress (by 43% and 71%, respectively) (Figure 4a and Figure 5e). In contrast, the inoculation with PGPB increased Mg (by 19–67%) and Fe contents in the roots (by 48–68%) compared with the control. Ni stress increased zinc (Zn) content in the roots by 26% (Figure 4b). The contents of manganese (Mn) and boron (B) in wheat roots decreased by 42 and 40% under Ni exposure. PGPB increased the contents of these elements by 14–22% compared with the control.

The negative effect of Ni stress was strongly evident, resulting in the reduction in copper (Cu) content in the roots of 89% (Figure 4d). AFI1 and AFI2 strains increased this value by 36–44%. Ni had similar effects on N, P, and Ca levels in the roots (Figure 5). The co-inoculation with PGPB maximized the levels of these elements. Potassium (K) content was increased significantly under Ni exposure. Bacterial inoculation promoted an increase in K content in the roots as well (Figure 5c).

Ni exposure reduced Si content in the straw by 35%, but the inoculation with the strain AFI2 increased Si content by 22% and the inoculation with both strains AFI1 and AFI2 by 18% (Figure 6f).

The inoculation with PGPB increased Mg content in wheat straw under Ni exposure by 66–84% as well as Cu by 16–28%, and N by 22–36% (Figure 6d and Figure 7a,e).

Bacteria significantly affected the accumulation of Mg in wheat straw, increasing the content of this element by three or more times. K content in the straw was 1.8-fold increased under Ni exposure. Bacteria also increased K by 7–12% under Ni exposure. Ca content in the straw decreased by 9% under Ni stress, and bacteria increased Ca by 3–10%. It was found that the Fe content was decreased by 33% under the influence of Ni, but PGPB AFI1 increased iron by 50%, AFI2 by 38%, and both strains together by 47% (Figure 6a).

Zn and Mn contents in wheat straw increased by 2.1 and 1.5 times under Ni exposure (Figure 6b,c). The effect of PGPB was significant for Zn, and nonsignificant for Mg. The B content in straw decreased significantly (by 53%) under Ni stress. Bacteria AFI2 increased the B content by 94%, and joint inoculation increased boron by 62% (Figure 6e).

Phosphorus decreased by 27% in the straw under Ni exposure. The inoculation with AFI1 and AFI2 strains increased P content by 59% and 11%, accordingly (Figure 7b). PGPB stimulated the accumulation of nitrogen in wheat straw (by 8–11%) in control conditions and under Ni exposure (by 10–15%). Thus, differences in the effect of PGPB on the content of P and B in wheat straw were expressed to a greater extent compared with the content of other elements (Figure S2).

The changes in the elemental composition of grains differed from those in the straw under Ni stress. Thus, the addition of Ni led to the accumulation of Zn, Cu and Mn in the grains by 0.8, 2.1 and 3.1 times more than the content of the corresponding elements in the control grains (Figure 8b–d).

The contents of Si, Fe, B, N, and P in wheat grains decreased under the influence of Ni (Figure 8). Si content in the grains decreased significantly (by 43%) under Ni stress; however, strain AFI2 increased Si content by 12% (Figure 8f). Mg content in the wheat grains was increased by 83% under Ni exposure (Figure 9e). PGPB also increased Mg content in the wheat grains (AFI1 by 56%, AFI2 by 40%).

Iron content was decreased by 40% under Ni exposure. The inoculation with AFI1 greatly increased the concentration of iron by 53%, with AFI2 by 12%, and with both strains by 30%. Zn and Mn contents increased significantly in the grains by three times, as well as in the straw under Ni exposure. PGPB contributed to an additional increase in the content of Zn and Mn by 6–16% (Figure 8a–c).

If a small outflow of Cu was observed in the straw during Ni stress, on the contrary, Cu was accumulated two times more in the grains (Figure 8d).The inoculation with PGPB slightly increased the concentration of Cu in the grains by 7–14%, and also increased the accumulation of B by 2–3 times. Strain AFI2 slightly increased N in grains (by 9%) under Ni stress (Figure 9a).

The main part of the phosphorus accumulated by plants is contained in wheat grains (Figure 9b). Phosphorus content in grains exceeds its content in straw by 5–10 times. The inoculation with PGPB under control conditions and Ni exposure did not affect P accumulation, with the exception of the variant with the inoculation with the AFI1 strain under Ni stress (increased by 24%). The effect of PGPB on the K content was mainly recorded in the straw, while minor changes were noted in grains. Also, very little Ca was contained in the grains, and it was mainly accumulated in the straw. AFI1 strain increased the Ca content in the grains by 10%. Differences were found in the effect of AFI1 and AFI2 strains on the content of elements against the background of Ni stress. This manifested in the content of such elements as N, P, Si, Fe, Zn, and Ca (Figures S2–S4).

### 2.5. Effect of Ni and PGPB on the Content of Water-Soluble (Mobile) Fractions of Elements at the End of the Experiments

Ni in a mobile form remained in high concentrations in the soil under Ni exposure at the end of experiments. In variants with PGPB inoculation, Ni (available to plants) significantly decreased by 11–18% (Table S4). The inoculation with PGPB contributed to the binding of Ni^2+^ and its transformation to insoluble forms inaccessible to the plants.

The inoculation with PGPB did not lead to a significant increase in the content of mobile phosphorus, with the exception of the inoculation with AFI1 strain under Ni stress. The contents of nitrate and ammonium nitrogen in the soil were increased by 95% and 33% respectively. The inoculation with PGPB had no significant effect on the content of nitrate and ammonium nitrogen. An increase in the potassium content available to plants by 28% was noted under Ni exposure. When PGPB were applied, K_2_O content was significantly decreased in the soil.

## 3. Discussion

During plant growth and development under abiotic stress (including Ni stress), a plant–microbe association is formed, comprising a variety of microorganisms attracted by the host plant to co-survive stress conditions [24]. This is confirmed by studies of microbial communities under stress. For example, the species diversity of the microbial community in the rhizosphere of plants under HM stress is dominated by microorganisms producing siderophores capable of binding HM ions [34]. When plants are inoculated with bacteria, an artificially created plant–bacteria association is formed. In such associations bacteria can migrate to various ecological niches of the plant to survive and protect their partner plant under the heavy metal stress [35].

Bacteria *Bacillus megaterium* AFI1 were found to be more tolerant to high Ni concentrations in liquid LB medium. Bacteria of the genus *Bacillus* are known to be tolerant to high Ni concentrations and can absorb Ni ions contributing to plant protection from Ni stress [11,21].

Improvement of plant mineral nutrition in the presence of PGPB occurs when micro- and macronutrients are converted into an accessible form due to changes in pH, enzymatic reactions and the production of acidic polysaccharides and organic and inorganic acids. Plants are known to increase the activity of antioxidant enzymes in response to stress caused by high concentrations of HMs [36,37,38,39]. This defense is activated at the transcriptional level, and enzymatic activity contributes to plant adaptation to Ni toxicity. Under Ni stress conditions in our experiments, the activities of POX and CAT enzymes were increased. Activation of POX and CAT under HM stress conditions leads to a reduction in the degree of oxidative damage to cells [36,37]. The strains AFI1 and AFI2 under Ni exposure decreased H_2_O_2_ production compared to the control level, indicating stress reduction in wheat plants. The accumulation of MDA, a marker of cell membrane damage, also decreased when PGPB were inoculated.

It should be noted that inoculation with PGPB can lead to various changes in the activity of antioxidant enzymes at different stages of wheat development. For example, strains AFI1 and AFI2 increased the POX activity of 14-day-old wheat plants cv. Leningradskaya 6 under mild Ni stress in hydroponic conditions [11]. Additionally, when winter wheat plants were inoculated with *Bacillus subtilis* BM2 bacteria under severe Ni stress, the activities of CAT, superoxide oxidase (SOD), and glutathione reductase (GR) were decreased [33]. Recently it has been shown that the strains *Azospirillum picis* B-2897T and *Enterobacter ludwigii* 11Uz increase CAT activity and decrease MDA content [32,40], and at an earlier stage of development, Ni accumulated primarily in wheat leaves. At the ripening stage, Ni accumulated primarily in the grains, which is consistent with previously obtained results [41].

According to studies using a high concentration gradient, the highest Ni translocation coefficients (CTs) were observed at concentrations of 50 and 100 mg/kg of soil [42]. The inoculation with AFI1 and AFI2 strains in our experiments led to a decrease in Ni, CBA and CT under Ni exposure.

The concentrations of photosynthetic pigments in plants decreased significantly under Ni stress (Table 2). In the experimental conditions, it was found that the concentrations of chlorophyll a in wheat plants were 21% lower than in the control, which could be caused by a decrease in the Mg concentration under the influence of nickel. Chl *b* turned out to be more sensitive to the effects of Ni, as its concentration decreased by 33%. It is known that Ni significantly reduces the levels of plastocyanin and ferredoxin in the thylakoid membrane, inhibits the activity of PS-II, and reduces the activity of key enzymes involved in the Calvin cycle [43]. The inoculation with bacteria under stress conditions led to a significant increase in the concentration of Mg and photosynthetic pigments in wheat plants. In addition, inoculation with bacteria contributed to an increase in the ratio of total chlorophyll to carotenoids, which is an indicator of the physiological state during plant adaptation to detect stress-dependent changes [44].

Ni in high concentrations inhibited wheat growth (Table 3), which is confirmed by other researchers [28,30]. The inhibition of plant growth under Ni stress is associated with a violation of transpiration and water balance [45,46], a decrease in the rate of cell division [47,48] and a violation of nutrient absorption [49,50].

Auxins produced by PGPB are able to increase plant root and shoot growth [51]. It is known that auxin acts on gene expression through a family of functionally distinct transcription factors, namely the DNA-binding auxin response factors (ARFs) [52]. Different ARFs regulate soluble sugars, promote root development and maintain chlorophyll content under the drought stress conditions, helping plants to adapt themselves to these stresses [52]. IAA biosynthesis by PGPB helps in successful root colonization, which leads to decreases in the drought stress in different plants [53]. It was shown that PGPB from the genera *Azospirillum* [51] and *Enterobacter* [40] have effects on wheat root systems and improve drought and Ni stress tolerance in wheat plants.

The strains AFI1 and AFI2 produced exopolysaccharides which have positive impacts on the plants under Ni stress. The multivariate analyses confirmed strong positive correlations among EPS applications, growth, and physiological traits under HM and salinity stress [54].

An imbalance in the absorption of macro-/micronutrients can seriously disrupt the systematic status of physiological processes leading to plant growth suppression [50,55]. A significant decrease in the content of macronutrients N, P, and Ca and trace elements Si, Fe, Cu, and B was observed under Ni stress. According to the literature data, Ni at a concentration of 0.08 mM contributed to a significant decrease in N, P, K, Ca, and Mg in green wheat seedlings [56].

Nickel accumulated maximally in wheat roots, where Mg, Mn, Fe, and Cu cations were replaced by Ni cations. In this study, it was found that Cu ions are maximally replaced by Ni, since its content decreased by nine times compared to the control. In our experiments, the cations’ exchange capacity in wheat roots decreased in the range Cu > Fe > Mg > Mn. It was found that under Ni stress conditions, N content in all organs of wheat plants decreased in the next row: the roots, the straw and the grains. When wheat plants were inoculated with the N-fixing AFI2 strain, the N content significantly increased in the grains under control and Ni stress conditions.

It is known that PGPB may convert the insoluble form of macronutrients (N, P, and K) into a soluble form through various mechanisms such as nitrogen fixation, phosphate solubilization, and metal cation chelation, increasing their availability to plants [30,57]. Under the influence of inoculation of spring wheat with *Pseudomonas fluorescens* and *P. putida*, the content of ash elements (K, Mg, Fe, Mn) in grains increased [28], which is consistent with our data. The treatment with the phosphorus-solubilizing strain AFI1 improves the content of phosphorus in wheat plants under Ni stress. The AFI1 strain increased the P content in the grains by 24%, while the AFI2 strain did not lead to changes in the phosphorus content. It was found that strain AFI possessed strong P-mobilizing activity (Table 1). The increase in P content will contribute to plant protection, since P is not only one of the main elements of plant nutrition, but also a stress regulator [58,59]. P optimizes plant growth; it increases stress resistance by increasing the production of antioxidants, osmolytes, and stabilizing membranes. The lack of P causes specific stress responses, such as changes in the root structure and metabolic shifts, which confirms the need for a balanced P content to increase plant resistance [59].

The addition of Ni salt to the soil significantly reduced the pH of the soil solution (Table S4), which led to a greater mobilization of phosphates compared to the control variant.

The maximum quantitative changes in the elemental composition under the influence of Ni stress occurred in the roots, which mainly accumulated potassium (Figure S2). K plays a dual role under Ni stress. Firstly, under the influence of K, the water-retaining capacity of plant protoplasm increases, which reduces their rate of wilting under nickel stress [60]. Secondly, when K accumulates in the roots, an electrochemical gradient is created that prevents the accumulation of excessive amounts of Ni ions [61]. It is known that K homeostasis plays an important role in plant response to abiotic stresses [62]. A strong accumulation of K was observed precisely in the wheat genotypes with pronounced adaptation to drought conditions [63]. K accumulated in chickpea plants more than other osmolytes (free amine nitrogen, proline, sugars) during drought [64]. Under Ni stress, wheat plants become dehydrated, and as a result, potassium accumulates, mainly in the roots and straw, as shown by the results of our experiments. The inoculation with PGPB under control conditions did not significantly change the K content in various organs of wheat Leningradskaya 6 with the exception of the effect of strain AFI2 inoculation on the increase in the K content in plant grains.

The increase in K concentration in the soil under Ni exposure at the end of the experiment, which we observed (Table S4), may be associated with osmotic stress of plants, which provoked potassium leakage [65].

Mg and Ca, on the contrary, were contained to a greater extent in the straw under Ni stress, preventing disorders in tissue structures. Ni stress led to a decrease in Ca accumulation in wheat grains. The inoculation with AFI1 and AFI2 bacteria had a positive effect on Ca accumulation in plant straw. Ca was significantly accumulated in the grains only after the inoculation with the AFI1 strain. It is known that Ca enhances photosynthesis and chlorophyll content and increases stomatal conductivity of plants, which counteracts the toxic effects of Ni [66].

Under control conditions, Si is contained in the aboveground mass of the plant in greater quantities than in the roots. Under Ni exposure, an increase in Si content in the roots and a decrease in straw and grains were noted (Figures S2–S4).

The grains were less susceptible to changes in the composition of the elements, but there was a tendency to decrease the contents of Si and N and to increase the Mg content under Ni stress (Figure S4). Silicate-solubilizing strain AFI2 significantly increased Si content in wheat grains. Such silicate-solubilizing bacteria are capable of destroying the structures of polysilicon minerals by synthesizing organic acids and converting silicon into the forms accessible to plants. The silicate-solubilizing bacterium *Bacillus altitudinis* SSB4 increased the growth of cereal crops and the silicon content in plants [67]. Bacteria from genus *Paenibacillus* were also found to be capable of solubilizing silicates [68,69]. Silicon is known to play a key role in strengthening cell walls, increasing stress resistance, and improving the absorption of other nutrients (nitrogen, phosphorus, and potassium) [70,71].

It was demonstrated in our experiments that Ni stress led to an increase in Mn accumulation, which is consistent with the results of other researchers [42]. Mn participates in photosynthesis (splitting of water, electron transfer), activation of enzymes (oxidation, phosphorylation) and synthesis of organic substances (proteins, sugars, vitamins) [72]. Zn also accumulated (by more than 1.5 times) in all organs of Leningradskaya 6 wheat, which is consistent with the results obtained earlier [28]. Zinc in physiological concentrations regulates metabolic pathways in plants, such as nitrogen metabolism, lipid metabolism, homeostasis of reactive oxygen species (ROS), photosynthesis, secondary metabolism, and mineral homeostasis [73]. In our experiments an accumulation of Zn and Mg was observed in the row: roots ˂ straw ˂ grains. The effect of Ni on B content was most pronounced in the roots and the straw, where it decreased compared to the control. The impact of microbial communities on the imbalance and competition between nickel and other microelements during their entry into the plant has not been sufficiently studied. It was surprising that under conditions of high nickel ion concentrations, Zn elements that compete with nickel also actively accumulate in wheat straw and grains, and bacteria increase their entry. Perhaps the accumulation Zn in wheat plants under Ni exposure can be explained as an additional mechanism of plant adaptation to stress.

It is known that Zn increases the activity of antioxidant enzymes and reduces the content of H_2_O_2_, which ultimately reduces the accumulation of MDA. Zn competes with Ni ions and reduces its toxicity by reducing the absorption and translocation of Ni by plants [74]. Differences in the accumulation of Zn under Ni stress may be due to wheat genotypes [74]. The increase in Zn uptake under Ni exposure is due to an increase in the activity of Zn transporters in response to Zn deficiency due to Zn substitution with Ni at the physiological binding sites [74].

The effect of Ni on the B content was most pronounced in the roots and in the straw, where it decreased compared to the control. B content is decreased in the grains by 43% under Ni exposure. B is known to help plants adapt to various abiotic stresses, such as drought, salinity, and HM stress [75]. B improves cell integrity, osmoprotection (e.g., proline synthesis), and the antioxidant system under stresses [75]. The inoculation with AFI1 and AFI2 strains significantly increased the concentration of B in wheat plants, which is consistent with a decrease in the level of MDA accumulation and an increase in the activity of antioxidant enzymes.

The concentration of Cu ions decreased as much as possible in the roots under Ni stress, but increased in the grains compared to the control. Cu mainly accumulated in the grains of wheat plants when the level of Ni contamination was below 200 mg/kg [42], which is consistent with our data. The inoculation with AFI1 and AFI2 strains under Ni stress contributed to the accumulation of Cu in plant roots by 36–44%.

Ni stress had the greatest effect on reducing the Fe content in the roots. Fe is an ion antagonist to Ni ion, since these ions compete for absorption by the plant and Fe decreases with excessive accumulation of Ni ions. Changes in Fe concentrations were less pronounced in the straw and the grains (Figures S3 and S4). Fe is a component of many vital enzymes, including cytochrome and ferredoxin in the electron transport chain, and is thus necessary to perform a wide range of biological functions. Fe is also involved in the synthesis of chlorophyll and the maintenance of the structure and function of chloroplasts in plants [76]. In our experiments, the inoculation with the AFI1 strain, which actively produces siderophores, led to a significant accumulation of Fe in all organs of wheat, both under control and under Ni stress. With an excess of Fe, PGPB can regulate the absorption and the transport of Fe, as well as increase ferritin expression to improve its storage [77]. The secretion of siderophores and the absorption of bound organic Fe are characteristic of many bacteria in the soil [78,79]. Bacteria of the genera *Bacillus* and *Paenibacillus* play an important role in providing plants with Fe or in overcoming its deficiency [80,81]. It has been found that *Bacillus* spp. bacteria associated with wheat are able not only to improve iron nutrition, but also to protect plants from stress at toxic Fe concentrations by increasing the expression of ferritin-encoding genes [77].

Phytohormones, which are produced by bacteria, may also be involved in plant defense against Ni stress [24]. In particular, abscisic acid plays a key role in enhancing tolerance to various abiotic stresses. It triggers signaling cascades that modulate the activity of K^+^ channels and transporters. Interestingly, many signaling pathways are common to the K^+^ and Cl^−^/NO^3−^ counter ions transport systems [82]. The inoculation with the AFI2 strain, which produces high amounts of abscisic acid, could help reduce osmotic stress caused by Ni exposure [11]. Studies have shown that both strains possess 1-aminocyclopropane-1-carboxylate (ACC) deaminase. It is known that bacteria expressing ACC deaminase counteract excess ethylene by breaking down ACC (the immediate precursor of ethylene) into ammonia and α-ketobutyrate, which serve as a source of nitrogen and carbon metabolism for bacteria [83]. α-ketobutyrate reduces ethylene formation in plants and thus mitigates the effects of stress [83]. In pot and model field experiments, it was shown that bacteria possessing ACC deaminase contributed to the adaptation of various plants to nickel stress, such as wheat [32,40], rice [84], and corn [85].

Ethylene biosynthesis occurs via the methionine cycle: the amino acid methionine is converted to S-adenosyl-L-methionine (SAM) by SAM synthetase, after that converting to 1-aminocyclopropane-1-carboxylic acid (ACC) by ACC synthase (ACS), and finally converting to gaseous ethylene by oxidation of ACC by ACC oxidase (ACO) [86,87]. Both ACS and ACO are encoded by multigene families with distinct spatial and stress-induced expression patterns, making them crucial control points for the environmental and hormonal regulation of ethylene production. Drought stress upregulates ACS7 and ACO2 in roots via ABA (abscisic acid)- and ROS (reactive oxygen species)-mediated signaling pathways. Exposure to heavy metals such as cadmium increases the abundance of ACS transcripts via MAPK (mitogen-activated protein kinase) cascades and oxidative signaling [88].

The bacteria-producing AAC deaminase (ACCD) protected plants from different stresses, including Ni stress [89]. ACCD alone may not be as effective in protecting plants from stress, because biologicals with various PGPB activities are more effective in plant stress tolerance [90,91]. It is possible that, in addition to ACC deaminase activity, there are other strategies by which the plant can suppress its ethylene signaling pathway in response to bacterial exposure under Ni stress, which probably rely on bacterial IAA production or other hormonal signals.

The process by which bacteria regulate plant homeostasis under HM stress is quite complex and manifests itself through their influence on the expression of various plant genes. For example, bacteria can regulate the expression of key genes responsible for metal transport and those involved in its metabolism [92,93].

The responses of wheat varieties with varying tolerance to nickel stress to inoculation under stress conditions remain poorly understood. Research on this area using genomics and transcriptomics methods is currently relevant.

There is currently a significant need for reliable and environmentally friendly agricultural management strategies under abiotic stresses. Taking into account concerns of increasing contamination in agricultural soils with HMs, particularly Ni, application of various PGPB that help plants adapt to new environmental conditions is a relevant strategy for monitoring and promoting sustainable agriculture. Furthermore, it is necessary to test the yield of plants growing under HM exposure to prevent them from entering the food chain at elevated levels. Biologicals that significantly reduce HM accumulation in plants can be used to grow plants in soils with low levels of HM contamination.

To generalize the obtained results, the study should be repeated using other varieties and nickel-sensitive wheat genotypes.

It should be noted that due to the poorly understood issue of the influence of temperature and humidity on plant–microbial relationships in field experiments, we cannot generalize the results obtained in model experiments under controlled conditions for the entire ecosystem. Therefore, these experiments must be repeated in the field on Ni-contaminated soils.

Further study of the effects of PGPB on plants in plant–bacteria associations under Ni stress, using genomics, proteomics, and metabolomics as priority areas, could reveal many mechanisms of systemic interactions and facilitate the development of next-generation biologicals.

## 4. Materials and Methods

### 4.1. Bacterial Properties

PGPB *Bacillus megaterium* AFI1: The sequences were submitted to the NCBI databases with accession numbers MZ468613 (1554 bp) for *Bacillus megaterium* AFI1 (the new species name for *Priestia megaterium*) and MZ468614 (1552 bp) for *Paenibacillus nicotianae* AFI2. Biochemical and physiological characteristics of these strains’ characteristics are given in the paper [11].

The strains *Bacillus megaterium* AFI1 and *Paenibacillus nicotianae* AFI2 were selected for vegetation experiments due to their ability to protect wheat plants from exposure to Ni^2+^ ions in minimal toxic concentrations [11]. The growth of bacteria on LB media (Sigma-Aldrich, St. Louis, MO, USA) containing various concentrations of Ni^2+^ (50 and 100) mg/L was studied. Ni^2+^ was added to the medium in the form of NiSO_4_ salt. The bacteria were added to the flasks with 50 mL LB medium and cultivated for 3 days (72 h) at 28 °C on Environmental Shaker Incubator ES 20/60 (Biosan, Riga, Latvia) at 180 rpm. Then, bacterial titer was determined, followed by centrifugal harvesting of supernatant. The centrifugation was carried out at 13,000 rpm on the Mini Spin Eppendorf centrifuge (Eppendorf SE, Gamburg, Germany). Ni content in supernatant and control variants (LB medium with addition of Ni^2+^ in 50 and 100 mg/L without bacterial inoculation) was determined by method of atomic absorption spectrometry (AA 240FS, Penang, Malaysia) with acetylene-air flame atomization.

#### 4.1.1. ACC Deaminase

The ACC deaminase was studied according to the protocol described in the article [94]. Dworkin and Foster (DF) minimal salts growth medium contained in g/L: glucose, 2 g; gluconic acid, 2 g; citric acid, 2 g; KH_2_PO_4_, 4 g; Na_2_HPO_4_, 6 g; MgSO_4_ · H_2_O, 0.2 g. CaCl_2_, 200 mg; FeSO_4_·7H_2_O, 200 mg; H_3_BO_3_, 15 mg; ZnSO_4_ 7H_2_O, 20 mg; Na_2_MoO_4_, 10 mg; KI, 10 mg; NaBr, 10 mg; MnCl_2_, 10 mg; COCl_2_, 5 mg; CuCl_2_, 5 mg; AlCl_3_, 2 mg; NiSO_4_, 2 mg supplemented with 3 mM ACC as the sole nitrogen source. Plates containing only DF minimal salts medium without ACC were used as the negative control and those with (NH_4_)_2_SO_4_ (0.2% *w*/*v*) were used as the positive control.

#### 4.1.2. Siderophore Production

The efficient method for determining siderophore production in bacteria cultured on solid media was used to study siderophore production traits within microbial populations [95]. A measurement of 0.061 g of CAS (chromazurol S) was diluted in 50 mL of water, and 10 mL of Fe(III) was added (1 mmol FeCl_3_·6H_2_O, 10 mmol HCl). A measurement of 0.073 g of HDTMA (hexadecyltrimethyl ammonium) was diluted in 40 mL of water. Pour CAS with iron slowly while stirring to HDTMA. A measurement of 30.24 g of HEPES (4-(2-hydroxyethyl)-1-piperazineethanesulfonic acid) was dissolved in 750 mL of water and 100 mL of concentrated CAS medium was added. Add 10 mL of sterile 20% glucose solution. Pour the resulting CAS agar into Petri dishes. The studied strains were sown on CAS agar in the center of Petri dishes. PGPB were incubated for 7 days at 28 °C. The ability to produce siderophores was determined by the presence of a colored zone (yellow or pink) around colony growth.

#### 4.1.3. Exopolysaccharides Assay

The Congo red agar assay was used to identify the presence of exopolysaccharides which are produced by the bacterial strain [96]. Assay plates were prepared by mixing in LB broth (25 g L^−1^), agar (20 g L^−1^), sucrose (10 g L^−1^), and Congo red (0.8 g L^−1^). All chemicals were well mixed and then autoclaved. Petri dishes with Congo red agar were inoculated with the strains studied. PGPRs grow for 5 days at 30 °C. The black colonies are visible against the red background, indicating the presence of polysaccharides [97].

#### 4.1.4. Indole Production Activity

Indole production activity (IPA) was assigned used Salkowski reagent (Gordon et al., 1951) [98]. The studied bacteria were grown in 100 mL R2A medium (Thermo Fisher Scientific, Waltham, MA, USA) with 500 mg/L tryptophan for 72 h at 28 °C at 180 rpm. Bacterial cells were removed by centrifugation during 90 s at 13,500 on Eppendorf AG 5804 R (Hamburg, Germany) for 15 min at 5000 rpm and the supernatant fluid was subsequently filtered through a 0.22 µm membrane filter. Then, 1 mL Salkowski reagent (1 mL 0.5 M FeCl_3_ in 50 mL of 35% HClO_4_) was added to 0.5 mL of supernatant. After 30 min of storage in the dark, the absorbances of colored supernatants were measured on a spectrophotometer Biomate 160 (Thermo Scientific™, Madison, WI, USA), at 540 nm. The indole contents were calculated used IAA standard curve based on dilution series of authentic IAA (0.5; 1.0; 5.0; 50.0 µg/mL).

#### 4.1.5. Phosphate-Solubilizing Activity

The ability of isolated bacteria to dissolve tricalcium phosphate was assayed on Pikovskaya medium as follows: glucose, 10 g; MnSO_4_·7H_2_O, 0.5 g; FeSO_4_·7H_2_O, 0.5 g; (NH_4_)_2_SO_4_, 0.5 g; NaCl, 0.2 g; KCl, 0.2 g; MgSO4·7H_2_O, 0.1 g; yeast extract, 0.5 g; 3.0 g of Ca_3_(PO_4_)_2_; and 20 g of agar in 1 L of distilled water (pH 7.2). The nutrient medium was poured into Petri dishes and tested strains were spread on Petri dishes. The Petri dishes were incubated at a temperature of 28 °C for 120–216 h. The phosphorus-solubilizing activity was judged as the appearance of clear zones around the growth area of a bacterial colony.

#### 4.1.6. Si- and Zn-Solubilizing Activities

For the determination of Si- and Zn-solubilizing activities, the bacteria were sown in Petri dishes with the Bunt and Rovira agar medium, containing (g L^−1^): glucose,10; ammonium sulphate, 1; potassium chloride, 0.2; di-potassium hydrogen phosphate, 0.1; magnesium sulphate, 0.2; agar 15, and pH 7.0. ZnO and SiO_2_ were added to final concentrations of 0.1%. Clear zones from each isolate were considered as positive reactions.

#### 4.1.7. Bacterial N-Fixing, Amylase, Protease, Lipase and Cellulase Activities

Nitrogen-fixing activity was assayed on the bacterial growth on nitrogen-free Ashby medium (sucrose—20.0 g, K_2_HP0_4_, 0.2 g; MgSO_4_·7H_2_O, 0.2 g; NaCl, 0.2 g; K_2_SO_4_, 0.2 g; CaCO_3,_ 5.0 g; agar, 18.0 g in 1 L of distilled water). The bacteria were incubated at a temperature of 28 °C for 48 h. Growth on the Ashby medium was assayed as positive test.

To study the amylase activity the medium of the following composition was used (peptone, 10 g; starch, 2.0 g; KH_2_PO_4,_ 0.3 g; MgSO_4_ 7H_2_O, 0.1 g; agar, 20g in 1 L of distilled water). The strains were inoculated into the center of the Petri dish by injection and Petri dishes were incubated for 5 days at a temperature of 28° C. The determination of amylase activity was done after adding the solution of potassium iodide to a Petri dish.

To study the protease activity, the following medium was used (casein, 20; glucose, 0.5; starch, 0.5; K_2_HPO_4_, 0.3; MgSO_4_ ·7H_2_O, 0.1; agar, 20 g/L of distilled water). The strains were inoculated into the center of a Petri dish by injection and incubated during 5 days at a temperature of 28° C. Then, 10% solution of trichloracetic acid was added to Petri dishes with grown strain. The protease activity of the strain was assessed by the hydrolysis zones of casein or their absence.

To study the lipase activity, a synthetic Seliber medium was used (g/L H_2_O: K_2_HPO_4_, 1; MgSO_4_ 7H_2_O, 0.3; CaCl_2_, 0.3; (NH_4_)_2_HPO_4,_ 1.5; NaCl, 0.3; agar—15; 1 mL of 1.6% aqueous solution of bromothymol blue in 1 L of distilled water). Then, 100 µL of sterile sunflower oil was distributed over the surface of the medium in a thin layer. The tested strains were inoculated into the center of a Petri dish by injection. After 5 days of cultivation at a temperature of 28 °C, hydrolytic digestion of the oil was noted by a change in the color of the indicator from blue to yellow.

Cellulase activity was assayed as the appearance of clear zones around the growth of a bacterium on Getchinson medium. The composition of the microbiological medium was as follows: (NaNO_3_, 2.5 g; K_2_HPO_4_, 1.0 g; MgSO_4_·7H_2_O, 0.3 g; NaCl, 0.1 g; CaCl_2_, 0.1 g; FeCl_3_, 0.01 g; yeast extract—0.1 g; microcrystalline cellulose—5.0 g; agar—20.0 g in 1 L of distilled water). Bacteria were incubated at a temperature of 28° C for 48 h. The estimation of cellulose activity was determined after adding a solution of potassium iodide to a Petri dish.

### 4.2. Plant Growth and Experimental Design

The object of the study was spring soft wheat (*Triticum aestivum* L.) cv. Leningradskaya 6 (resistant to high Ni^2+^ concentrations). The experimental scheme included variants with inoculation with bacterial strains *B. megaterium* AFI1, *P. nicotianae* AFI2 and both strains under Ni stress and also in control conditions. The experiments used sod-podzolic light loamy soil with the following parameters: C org, 1.8%; P_2_O_5,_ 198 mg/kg; K_2_O, 112 mg/kg; N-NO_3,_ 18.2 mg/kg and N-NH4, 34.6 mg/kg; pH (KCl), 6.34. The soil was characterized by the following granulometric composition of fractions, %: 1–0.25 mm, 2.6; 0.25–0.05 mm, 14.3; 0.05–0.01 mm, 45.6; 0.01–0.005 mm, 9.1; 0.005–0.001 mm, 12.0, <0.001 mm, 16.4. The density of the solid phase of the soil was 2.55 g/cm^3^.

The plants were grown in vegetative lighting installations with the following parameters: illumination with DNaZ-400 lamps (Reflax, Moscow, Russia), the illumination intensity 30–35 kLux, photoperiod of 16 h, temperatures 23–24 °C (day temperatures) and 19–20 °C (night temperatures), and air humidity 70–80%. For variants with Ni stress, the solution of NiCl_2_·6H_2_O (at the concentration of Ni^2+^ 100 mg/kg of soil) was added by stirring the soil. The moisture content of the soil was maintained at 60% of the total moisture capacity.

Wheat seeds were washed with tap water and distilled water after the surface sterilization in 70% ethanol for 2 min. Then, seeds were placed in Petri dishes for the germination in a thermostat at 26 °C for 48 h. The germinated seeds were planted in 4 liter pots (D = 20 cm, H = 18 cm) (5 plants per pot) with sod-podzolic light loamy soil. Wheat plants were inoculated with bacterial strains *B. megaterium* AFI1 and *P. nicotianae* AFI2 three times at the time of planting and at the wheat growth stages of Z 05 (germination), Z 21 (tillering) and Z 30–31 (stem elongation) according to [99]. A suspension of bacterial cells at the rate of 1 mL (10^8^ CFU/mL) per seedling was applied to the soil surface around each plant at growth stage Z05. Control plants were inoculated with 1 mL sterilized growth bacterial medium LB. At stages Z 21 and Z 30–31, spraying was carried out with bacterial strains at a concentration of 10^5^ CFU/mL. The control variant was sprayed with LB medium. Irrigation with water was carried out daily (to 65% soil water capacity). The experiment was conducted using a Randomized Complete Block Design (RCBD) with five treatments with three replicates. The experiment was carried out 2 times. Leaf sampling for biochemical analysis was collected at the stage of initial tube elongation (Z 30). The contents of chlorophylls (chl *a* and chl *b*), carotenoids (car), malondialdehyde (MDA), and hydrogen peroxide (H_2_O_2_), as well as the activities of catalase (CAT) and peroxidase (POX), were determined at initial tube elongation (Z 30) stage. The detection of ash elements in plant material was done after harvesting.

The plants were harvested at 80 days after sowing at stage Z 90. The roots were rinsed from the soil with tap water and then distilled water. The plants (straw together with grain) and roots after cutting the plants were dried at 70 °C to a constant weight and weighed.

### 4.3. Analysis of Plant Materials

#### 4.3.1. Chlorophyll and Carotenoids Analysis in Plants

The determination of chlorophyll a (chl *a*), chlorophyll b (chl *b*), and carotenoids (car) concentrations was done as described previously [100].

The fresh leaves (0.2 g) were ground in a porcelain mortar with a small amount of acetone and sand. The ground mass was placed in the tubes containing 10 mL of 80% acetone for 24 h at 20 °C. The tubes with plant ground mass were centrifuged at 20,000× *g* for 20 min. Then, supernatant was transferred into a 50 mL volumetric flask and made up to the mark with acetone. The absorbance of the resulting supernatant was measured at 663, 645 and 470 nm using a spectrophotometer (Model Spekol 1500, Jena, Germany). The pigment concentrations in mg/dm^3^ were calculated using the following formulas [100]:

C chl *a* (mg/dm^3^) = (9.784 × OD662) − (0.99 × OD644)

C chl *b* (mg/dm^3^) = (21.426 × OD644) − (4.65 × OD662)

C car (mg/dm^3^) = (4.695 × OD440.5) − (0.268 (C chl *a* + C chl *b*)

To recalculate the pigments concentrations X in mg/g the following formula was used:

X = C × V/n × 1000

where C—pigment content (mg/dm^3^), V—extract volume in cm^3^, and n—sample weight in grams.

#### 4.3.2. Malondialdehyde (MDA) Content

The malondialdehyde (MDA) content was measured using the method described in [101]. A measurement of 0.3 g of fresh leaves was homogenized and filtered. The reaction medium consists of 0.3 mL plants homogenate, 3 mL 1% H_3_PO_4_ and 1 mL 0.6% thiobarbituric acid (TBA) aqueous solution. The homogenate was put in a water bath at 95–100 °C for 60 min, after which the samples were cooled down. Then, 4 mL of n-butanol was added and centrifuged for 10 min at 10,000× *g*. The absorbance of butanol extract was measured at 532 nm and 600 nm using the spectrophotometer (Model Spekol 1500, Jena, Germany). The concentration of TBA-reactive products was expressed in MDA µM g^−1^ FW.

#### 4.3.3. Catalase (CAT)

The CAT was measured using the method described in [102]. This method is based on the effect of CAT on hydrogen peroxide (H_2_O_2_) and the measurement of the ultraviolet absorption of H_2_O_2_ at 240 nm. The reaction mixture at a volume of 3 mL contained 0.1 M of sodium phosphate buffer (pH 7.0), 2 mM of H_2_O_2_ and 0.2 mL of enzyme extract. For the calculation of the activity, the extinction coefficient of 0.036 mM^−1^ cm^−1^ was employed. CAT activity was expressed in µM of H_2_O_2_ per mg^−1^ protein min^−1^.

#### 4.3.4. POX Activity

Activity of POX in plants was determined as described previously [103].

A 200 mg weight of plant material is finely ground in a porcelain mortar with acetate buffer with pH 5.4 and transferred into a 50 cm^3^ measuring flask. After 10 min of infusion the extract is centrifuged at 4000 rpm. Two quartz cuvettes with a working length of 20 mm are filled with 2 cm^3^ of centrifugate and 2 cm^3^ of benzidine. The cuvettes in the photoelectric colorimeter are placed against the red filters (590 nm). At the beginning with the left drum set the galvanometer pointer to zero, then with the right drum move the galvanometer pointer to the leftmost position (D = 0.125 or 0.250). After that, 2 cm^3^ of water is poured into the control cuvette, and 2 cm^3^ of 0.3% hydrogen peroxide is poured into the measuring cuvette. When the first drop of hydrogen peroxide is added, the stopwatch is started. The galvanometer hand starts to deviate from the edge of the scale to the zero division. The stopwatch is stopped when the galvanometer hand reaches zero. From the reaction rate found, the POX activity (A) is calculated, using the following formulas.

*A = D × a × b × c/f × t*

*D*—optical density, equal to 0.125 or 0.250;
*a*—ratio of the amount of liquid taken for preparation of the extract to the mass of raw tissue, cm^3^/g;
*b*—degree of additional dilution of the extract after centrifugation;
*c*—degree of constant dilution of the extract in the reaction mixture in the cuvette;
*f*—layer thickness (2 cm);
*t*—time, s.

POX activity was expressed in units, each representing the absorbance value per second per g^−1^ FW.

#### 4.3.5. Determination of Hydrogen Peroxide in Plants

To determine H_2_O_2_ in plants, leaf extraction was performed using chilled acetone, in a ratio 0.3 (g) of the sample with 1.5 mL of acetone 100%. The sample was centrifuged for 10 min (at 13,000 rpm). A measurement of 0.5 mL of centrifuge was stained with 0.5 mL of xylene orange reagent. Light absorption was measured on a spectrophotometer (PE-3000UF, Shanghai, China).

#### 4.3.6. Detection of Ash Elements (K, P, Ca, Mg, Ni, Fe, Zn, Mn, SiO_2_, and B) in Plant Material

Ni and other element contents in wheat shoots and roots were determined by method of atomic absorption spectrometry (AA 240FS, Penang, Malaysia) with acetylene-air flame atomization. Dried 1 g samples of plant materials were burned in porcelain crucibles at 450 °C. The ash was dissolved in 2 cm^3^ of a 1:1 mixture of HCl and water with heating to boiling point. The solution was cooled and transferred with water to a 100 cm^3^ volumetric flask. The results were expressed as mg kg^−1^ of absolutely dry sample. Solutions of Ni salts of different concentrations were used to calibrate the atomic absorption spectrometer.

#### 4.3.7. Detection of Nitrogen in Plant Material

Nitrogen content in the plant material was detected by the indophenolic method. The method is based on the formation of a blue colored compound (indophenol) during the reaction of ammonia with phenol and sodium hypochlorite in the presence of sodium nitroprusside. A measurement of 0.2 g of plant material was placed in a test tube and 2 mL sulfuric acid with selenium (1 g selenium per 200 mL concentrate sulfuric acid) was added. The tube was heated until the solution was discolored. A measurement of 1 mL of the solution is transferred by a dispenser into a dry conical flask with a capacity of 100 mL. Measurements of 45 mL of coloring reagent and 2.5 mL of hypochlorite working solution are added to the solution with a dispenser. After adding each reagent, the solution is mixed. The flask with the solution is left for 1 h to form a stable color. The absorbance was measured in 1 cm cuvette at a wavelength of 655 nm (PE-3000UF, Shanghai, China).

### 4.4. Soil Analysis

After growing the plants mobile fractions of nickel, phosphorus, potassium, exchangeable ammonium, nitrates and soil pH were determined.

#### 4.4.1. Determination of Mobile Ni

To determine mobile nickel, 5 g of soil was poured into 50 mL of ammonium acetate buffer with a pH of 5.5, shaken for 30 min in a shaker (US-1350L, Wang Chai, China), filtered through a paper filter and spectrometered on an atomic absorption spectrophotometer (AA 240FS, Penang, Malaysia).

#### 4.4.2. Determination of Mobile P and K in the Soil

After the end of the experiment, mobile phosphorus and potassium compounds in the soil were determined by the Kirsanov method in an extract of 0.2 M/L hydrochloric acid with a soil–solution ratio of 1:5 when mixed for 1 min. After filtration, 38 mL of antimony–molybdenum reagent in 5 M sulfuric acid was added to 2 mL of the extract and the corresponding calibration solutions. The reagent was prepared by mixing 200 mL of a 3% solution of ammonium molybdate, 100 mL of a 0.15% solution of potassium antimony, 500 mL of 5 M/L sulfuric acid and 200 mL of distilled water. Then, 1 g of ascorbic acid is added to 170 mL of the resulting mixture and the volume is adjusted to 1 L of distilled water. After 10 min, the absorbance of the solution was determined by spectrophotometry at a wavelength of 710 nm and 1 cm cuvette on a PE-3000UF spectrophotometer (China, Shanghai).

The potassium content in the extract was determined on a flame photometer (FPA–2–01, Sergiev Posad, Russia) using a light filter with maximum transmission in the range of 766–770 nm.

#### 4.4.3. Determination of Exchangeable NH_4_

Exchangeable ammonium was determined by displacing it from the soil with a solution of potassium chloride (1 mol/L) at a soil–solution ratio of 1:2.5 and staining the extract with an indophenol reagent. The reagent was prepared by dissolving 56.7 g of sodium salicylate, 16.7 g of potassium–sodium tartaric acid, and 26.7 g of sodium hydroxide in 700 mL of water and boiling for 20 min. A measurement of 0.4 g of sodium nitroprusside was added to the cooled solution and, after its dissolution, the volume of the reagent is reduced to 1 liter. Before use, the reagent is diluted with distilled water in a ratio of 1:9 and 0.2% trilon EDTA-N was added to the solution. Then, 40 cm^3^ of the coloring reagent is added to 2 cm^3^ of the filtered extract (and calibration solutions). After that, 2 cm^3^ of 0.125% sodium hypochlorite was added. Then, the absorbance of the colored solutions was measured with 1 cm cuvette at a wavelength of 655 nm after 1 h (PE-3000UF, Shanghai, China).

#### 4.4.4. Determination of N-NO3 and pH

Nitrates were determined in the extract using an ion-selective electrode (ECOM–NO3, Moscow, Russia) and the pH meter Ecotest-120 (Econix, Russia, Moscow) after extraction with a solution of potassium sulfate with a concentration of 1 M/L with a ratio of soil mass and solution volume of 1:2.5.

After growing the plants, the pH of the soil suspension was determined in 1 M KCl solution (soil solution ratio 1:2.5) using the Ecotest-120 pH meter ionomer (Econix, Russia, Moscow).

### 4.5. Statistical Analysis

The independent experiments were performed twice to assess the wheat growth parameters. We used three biological replicates, with 5 wheat plants per replicate (*n* = 15), to assess the biochemical parameters of plants (*n* = 3). The data were statistically evaluated using STATISTICA-11 and subjected to a two-way analysis of variance (ANOVA). The mean values are shown as error bars representing standard errors of the means in all the figures. The data are presented as average mean standard error (SEM). Duncan’s multiple range test was performed to determine significant differences between individual means. The differences between the means were determined at the level of significance (*p* < 0.95).

## 5. Conclusions

Ni at a high concentration (100 mg/kg) in the soil leads to an increase in the content of Ni and K in wheat plants cv. Leningradskaya 6. Ni reduces the content of individual elements of mineral nutrition, which affects the development of wheat plants. The contents of Si, Fe, B, N, and P in wheat grains decreased under Ni exposure. *Bacillus megaterium* AFI1 significantly reduced the Ni concentration in wheat grains due to partial binding of Ni ions by endogenous polysaccharides and siderophores. PGPB *Bacillus megaterium* AFI1 and *Paenibacillus nicotianae* AFI2 increased antioxidant protection of plants by decreasing the level of stress ethylene and regulating the homeostasis of nutrients in wheat plants. The phosphate-solubilizing strain AFI1 improved the content of P in wheat plants under Ni exposure. N-fixing and silicate-solubilizing strain *Paenibacillus nicotianae* AFI2 increased N and Si contents. Both strains helped wheat plants cope with Ni stress.

## Figures and Tables

**Table 1:** Metabolic activities of PGPB.

Strain	ACC	AA	PM	PP	LA	SA	SP	IP	PA	NA	AP	ZS
*B. megaterium* AFI1	++	+	++	++	++	-	++	++	+	-	-	+
*P. nicotianae* AFI2	+	+	-	+	+	+	-	+	+	+	-	-

Notes: **ACC**: ACC deaminase activity; **AA**: Amylolytic activity; **PM**: Phosphate-mobilizing activity; **PP**: Polysaccharides production; **LA**: Lypolytic activity; **SA**: Silicate-solubilizing activity; **SP**: Siderophore production; **IP**: Indole production; **PA**: Proteolytic activity; **NA**: Nitrogen-fixing activity; **AP**: Ammonium (NH_4_) production; **ZS**: Zinc-solubilizing activity; ++ strong activity, + medium and weak activity, - no activity.

**Table 2:** The effect of PGPB *Bacillus megaterium*AFI1 and *Paenibacillus nicotianae* AFI2 on the antioxidant activity of wheat cv. Leningradskaya 6 under Ni exposure.

Variant	MDA, mcM/g	POX, Units/g FW	CAT, µM of H_2_O_2_ per mg^−1^ Protein min^−1^	Ni, mg/kg	H_2_O_2_ mcM/g
Control	3.84 ± 0.26 ^a^	45.9 ± 3.2 ^ab^	484.9 ± 15.2 ^b^	2.36 ± 0.18 ^a^	2.65 ± 0.18 ^a^
AFI1	3.51 ± 0.22 ^ab^	50.6 ± 3.9 ^a^	549.4 ± 17.2 ^a^	0.53 ± 0.02 ^c^	2.46 ± 0.16 ^a^
AFI2	3.74 ± 0.26 ^a^	51.3 ± 3.8 ^a^	542.7 ± 17.0 ^a^	1.81 ± 0.16 ^b^	2.07 ± 0.12 ^b^
AFI1 + AFI2	3.24 ± 0.19 ^b^	52.0 ± 3.7 ^a^	534.4 ± 16.0 ^ab^	0.41 ± 0.02 ^d^	2.36 ± 0.16 ^ab^
Ni	5.03 ± 0.31 ^a^	60.4 ± 4.0 ^a^	616.8 ± 26.0 ^a^	52.18 ± 2.6 ^a^	4.25 ± 0.3 2 ^a^
Ni + AFI1	4.03 ± 0.27 ^b^	56.0 ± 3.9 ^ab^	592.0 ± 22.0 ^ab^	30.66 ± 1.2 ^c^	2.16 ± 0.12 ^b^
Ni + AFI2	4.12 ± 0.37 ^b^	56.5 ± 3.7 ^ab^	593.5 ± 20.8 ^ab^	40.98 ± 1.8 ^b^	1.95 ± 0.06 ^c^
Ni + AFI1 + AFI2	4.00 ± 0.34 ^b^	54.6 ± 3.5 ^b^	571.1 ± 16.0 ^b^	38.85 ± 1.7 ^bc^	2.26 ± 0.14 ^b^

Notes: Control: Non-inoculated wheat plants. Ni: The addition of 100 mg/kg Ni^2+^. AFI1 and AFI2: Wheat plants inoculated with appropriate strains *Bacillus megaterium* AFI1 and *Paenibacillus nicotianae* AFI2. Bars show ±SEM. Values in columns followed by different letters (a–d) are significantly different at the *p* ≤ 0.05, as determined by Duncan’s multiple range test.

**Table 3:** The effect of Ni and PGPB *Bacillus megaterium* AFI1 and *Paenibacillus nicotianae* AFI2 on the contents of photosynthetic pigments in the leaves of wheat cv. Leningradskaya 6.

Variant	Chl *a*, mg/100 g	Chl *b*, mg/100 g	Chl *a + b*, mg/100 g	Car, mg/100 g	Chl *a/b*	Chl *a + b*/Car
Control	128.8 ± 6.4 ^c^	41.3 ± 2.4 ^ab^	170.1 ± 9.6 ^c^	34.8 ± 2.3 ^c^	3.12	4.89
AFI1	125.3 ± 6.1 ^c^	35.6 ± 2.0 ^c^	160.9 ± 9.4 ^c^	40.1 ± 2.8 ^b^	3.52	4.01
AFI2	141.1 ± 6.2 ^b^	40.2 ± 2.8 ^b^	181.3 ± 10.4 ^b^	39.9 ± 2.7 ^b^	3.51	4.29
AFI1 + AFI2	159.1 ± 9.5 ^a^	46.0 ± 2.4 ^a^	205.1 ± 12.5 ^a^	50.3 ± 3.0 ^a^	3.46	4.08
Ni	102.1 ± 7.1 ^c^	27.7 ± 1.8 ^b^	129.9 ± 6.3 ^c^	33.2 ± 2.1 ^c^	3.69	3.91
Ni + AFI1	139.9 ± 7.8 ^b^	38.6 ± 2.1 ^ab^	178.6 ± 9.8 ^b^	43.8 ± 2.8 ^b^	3.62	4.08
Ni + AFI2	154.2 ± 7.7 ^a^	42.1 ± 2.5 ^a^	196.3 ± 11.3 ^a^	48.4 ± 3.1 ^a^	3.66	4.06
Ni + AFI1 + AFI2	156.6 ± 9.4 ^a^	41.4 ± 2.3 ^a^	198.1 ± 11.5 ^a^	51.1 ± 3.3 ^a^	3.78	3.88

Notes: Control: Non-inoculated wheat plants. Ni: The addition of 100 mg/kg Ni^2+^. AFI1 and AFI2: Wheat plants inoculated with appropriate strains *Bacillus megaterium* AFI1 and *Paenibacillus nicotianae* AFI2. Chl *a*—chlorophyll *a*, Chl *b*- chlorophyll *b*, Chl *a + b*—the sum of chl, car—carotenoids. Bars show ±SEM. Values in columns followed by different letters (a–c) are significantly different at the *p* ≤ 0.05, as determined by Duncan’s multiple range test.

**Table 4:** Effect of PGPB *Bacillus megaterium*AFI1 and *Paenibacillus nicotianae* AFI2 on the plant yield of wheat cv. Leningradskaya 6 (ripening stage) under Ni exposure.

Variant	Plant Yield (Dry Matter), g/pot		
	Grains	Straw	Roots
Control	7.14 ± 0.37 ^c^	14.14 ± 0.67 ^bc^	1.96 ± 0.08 ^c^
AFI1	8.96 ± 0.42 ^b^	16.72 ± 0.78 ^ab^	2.56 ± 0.11 ^ab^
AFI2	10.35 ± 0.48 ^a^	18.85 ± 0.68 ^a^	2.34 ± 0.12 ^b^
AFI1 + AFI2	9.38 ± 0.39 ^ab^	17.84 ± 0.92 ^a^	2.75 ± 0.09 ^a^
Ni	5.32 ± 0.22 ^d^	12.01 ± 0.54 ^d^	1.36 ± 0.05 ^d^
Ni + AFI1	7.56 ± 0.37 ^c^	14.91 ± 0.48 ^bc^	1.81 ± 0.08 ^c^
Ni + AFI2	7.28 ± 0.31 ^c^	15.22 ± 0.69 ^bc^	1.73 ± 0.09 ^c^
Ni + AFI1 + AFI2	7.84 ± 0.47 ^c^	15.67 ± 0.77 ^b^	1.92 ± 0.09 ^c^

Notes: Control: Non-inoculated wheat plants. Ni: The addition of 100 mg/kg Ni^2+^. AFI1 and AFI2: Wheat plants inoculated with appropriate strains *Bacillus megaterium* AFI1 and *Paenibacillus nicotianae* AFI2. Bars show ±SEM. Values in columns followed by different letters (a–d) are significantly different at the *p* ≤ 0.05, as determined by Duncan’s multiple range test.

## Data Availability

The data presented in this study are available upon request from the corresponding authors.

## Supplementary Materials

The following supporting information can be downloaded at: https://www.mdpi.com/article/10.3390/ijms27115041/s1.
