# Supplementary material for: PGPB Bacillus Megaterium AFI1 and Paenibacillus Nicotianae AFI2 Improve Nutrient Uptake and Stimulate Adaptation of Wheat Under Nickel Exposure
Source: Int J Mol Sci. 2026 Jun 2;27(11):5041. doi: 10.3390/ijms27115041 (PMC13256199; doi:10.3390/ijms27115041)
Supplement: Supplementary file 1 [file ijms-27-05041-s001.zip › ijms-4318151-supplementary.pdf]

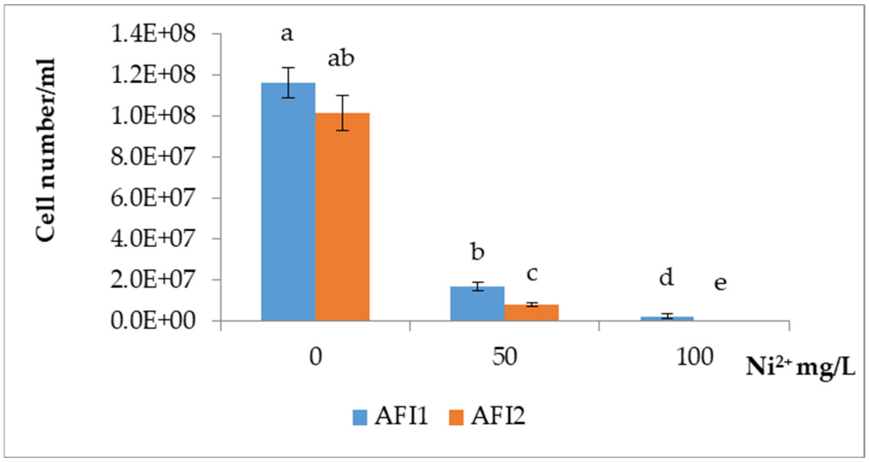

Figure S1. Growth of PGPB studied on LB medium with different concentrations of Ni^{2+}. Notes: different letters (a–e) are significantly different at the $p \leq 0.05$, as determined by Duncan's multiple range test. AFI1: *Bacillus megaterium* AFI1, AFI2: *Paenibacillus nicotianae* AFI2.

Table S1. The effect of Ni and PGPB *Bacillus megaterium* AFI1 and *Paenibacillus nicotianae*. AFI2 on the area of flag leaf of wheat cv. Leningradskaya 6.

Variant	Flag leaf length, cm	Flag leaf width, cm	Flag leaf area, cm²
Control	19.6±1.5 ^{bc}	0.68±0.03 ^{b}	8.9±0.4 ^{b}
AFI1	22.1±1.3 ^{ab}	0.76±0.04 ^{a}	11.2±0.4 ^{a}
AFI2	22.6±1.1 ^{a}	0.81±0.07 ^{a}	12.2±0.7 ^{a}
AFI1+AFI2	22.7±1.4 ^{a}	0.77±0.05 ^{a}	11.7±0.5 ^{a}
Ni	17.9±1.3 ^{c}	0.61±0.03 ^{c}	7.3±0.4 ^{c}
Ni+AFI1	20.0±0.9 ^{bc}	0.68±0.04 ^{b}	9.1±0.4 ^{b}
Ni+AFI2	20.7±1.4 ^{ab}	0.62±0.03 ^{bc}	8.6±0.6 ^{b}
Ni+AFI1+AFI2	20.3±1.1 ^{ab}	0.67±0.05 ^{b}	9.1±0.5 ^{b}

Notes: Control: non-inoculated wheat plants. Ni: the addition of 100 mg/kg Ni^{2+}. AFI1 and AFI2: wheat plants inoculated with appropriate strains *Bacillus megaterium* AFI1 and *Paenibacillus nicotianae* AFI2. Bars show ±SEM. Values in columns followed by different letters (a–c) are significantly different at the $p \leq 0.05$, as determined by Duncan's multiple range test.

Table S2. The **c**oefficients of Ni biological accumulation (CBA) and Ni translocation (CT) in wheat grains.

Variant	Ni	Ni+AFI1	Ni+AFI2	Ni+AFI1+AFI2
CBA, 1st year	0.166±0.009^{a}	0.111±0.006^{c}	0.129±0.007^{b}	0.127±0.007^{b}
CBA2, 2nd year	0.180±0.010^{a}	0.121±0.007^{c}	0.139±0.008^{b}	0.135±0.008^{b}
CBA average	0.173±0.009^{a}	0.116±0.006^{c}	0.134±0.006^{b}	0.131±0.006^{b}
CT, 1st year	0.098±0.006^{a}	0.073±0.004^{c}	0.083±0.005^{b}	0.083±0.005^{b}
CT 2nd year	0.101±0.006^{a}	0.075±0.004^{bc}	0.084±0.005^{b}	0.084±0.005^{b}
CBA average	0.099±0.004^{a}	0.074±0.003^{c}	0.084±0.003^{b}	0.083±0.003^{b}

Notes: Ni: non-inoculated wheat plants, growing under Ni stress (100 mg Ni^{2+} per 1 kg of soil). AFI1 and AFI2: wheat plants inoculated with appropriate strains *Bacillus megaterium* AFI1 and *Paenibacillus nicotianae* AFI2. CBA= Ni concentration in the grains/Ni concentration in the soil, CT= Ni concentration in the grains/Ni concentration in the roots. Bars with different letters in the rows are significantly different at $p \leq 0.05$, as determined by Duncan's multiple range test. Wheat plants cv. Leningradskaya 6 were grown under controlled conditions during two vegetation experiments (80 days).

Table S3. Coefficients of Ni biological accumulation and Ni translocation in wheat straw.

Variant	Ni	Ni+AFI1	Ni+AFI2	Ni+AFI1+AFI2
CBA, 1st year	0.131±0.007[a]	0.104±0.006[b]	0.118±0.007[ab]	0.111±0.006[b]
CBA2, 2nd year	0.166±0.009[a]	0.123±0.007[c]	0.145±0.008[b]	0.146±0.008[b]
CBA average	0.149±0.016[a]	0.113±0.009[b]	0.131±0.013[ab]	0.128±0.016[ab]
CT, 1st year	0.077±0.004[a]	0.069±0.004[ab]	0.076±0.004[a]	0.072±0.004[ab]
CT 2nd year	0.093±0.005[a]	0.077±0.004[b]	0.088±0.005[ab]	0.090±0.005[a]
CBA average	0.085±0.008[a]	0.073±0.004[ab]	0.082±0.006[a]	0.081±0.008[ab]

Notes:Ni: non-inoculated wheat plants, growing under Ni stress (100 mg Ni^{2+} per 1 kg of soil).AFI1 and AFI2: wheat plants inoculated with appropriate strains *Bacillus megaterium* AFI1 and *Paenibacillus nicotianae* AFI2. CBA - Ni concentration in the straw/Ni concentration in the soil, CT - Ni concentration in the straw/Ni concentration in the roots. Bars with different letters in the rows are significantly different at p ≤ 0.05, as determined by Duncan's multiple range test. Wheat plants cv. Leningradskaya 6 were grown under controlled conditions during two vegettation experiments (80 days).

Table S4. Effect of PGPB on the content of mobile fractions of elements in the soil at the end of the experiment.

Variant	pH_{KCl}	P_2O_5, mg/kg	K_2O, mg/kg	$N\text{-}NO_3$, mg/kg	$N\text{-}NH_4$, mg/kg	Ni, mg/kg
Control	6.2±0.1[a]	190.60±15.1[c]	65.90±5.2[bc]	26.30±2.1[d]	7.58±0.6[c]	0.8±0.1[c]
Ni	5.8±0.2[a]	214.30±17.0[b]	90.90±7.2[a]	51.30±4.1[b]	10.07±0.8[a]	93.81±7.4[a]
Ni +AFI1	5.8±0.1[a]	249.30±19.8 [a]	75.50±6.0[b]	61.70±4.9 [a]	10.29±0.8[a]	77.26±6.1[b]
Ni + AFI2	5.8±0.2[a]	204.40±16.2[bc]	59.90±4.7[c]	45.70±3.6[c]	9.50±0.8[ab]	83.43±6.6[b]
Ni+AFI1 +AFI2	5.8±0.2[a]	213.10±16.9[b]	71.10±5.6[bc]	47.70±3.8[bc]	8.58±0.7[b]	80.50±6.4[b]

Notes: Ni: non-inoculated wheat plants, growing under Ni stress (100 mg Ni^{2+} per 1 kg of soil).AFI1 and AFI2: wheat plants inoculated with appropriate strains *Bacillus megaterium* AFI1 and *Paenibacillus nicotianae* AFI2. Bars with different letters are significantly different at p ≤ 0.05, as determined by Duncan's multiple range test.

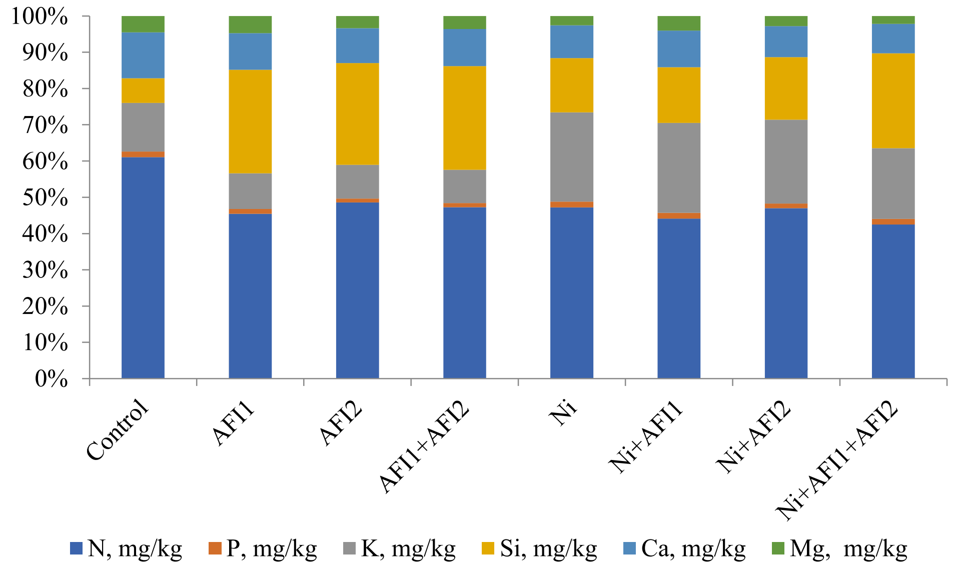

Figure S2. The effect of PGPB on relative content of macronutrients and silicon in wheat roots. Notes: **Control**: non-inoculated wheat cv. Leningradskaya 6. **Ni**: the addition of 100 mg/kg Ni^{2+}. **AFI1** and **AFI2**: wheat plants inoculated with appropriate strains *Bacillus megaterium* AFI1 and *Paenibacillus nicotinae* AFI2.

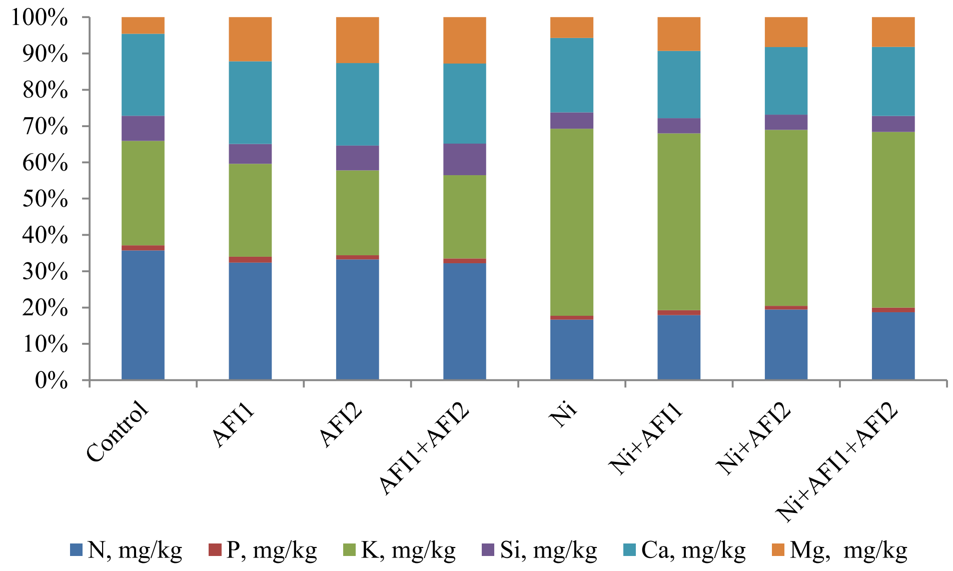

Figure S3. The effect of PGPB on relative content of macronutrients and silicon in wheat straw. Notes: Control: non-inoculated wheat cv. Leningradskaya 6. Ni: the addition of 100 mg/kg Ni2+. AFI1 and AFI2: wheat plants inoculated with appropriate strains *Bacillus megaterium* AFI1 and *Paenibacillus nicotinae* AFI2.

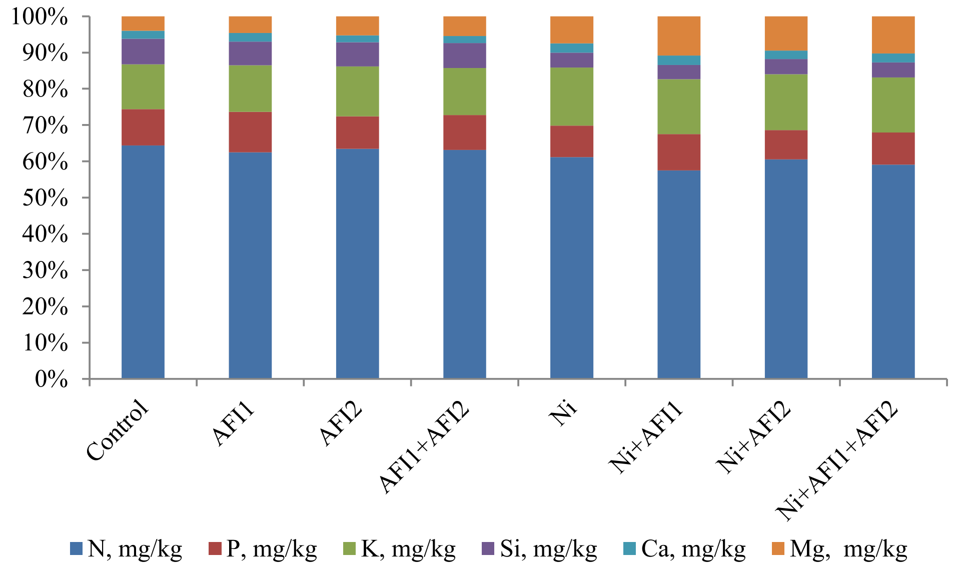

Figure S4. The effect of PGPB on relative content of macronutrients and silicon in wheat grains. Notes: Control: non-inoculated wheat cv. Leningradskaya 6. Ni: the addition of 100 mg/kg Ni2+. AFI1 and AFI2: wheat plants inoculated with appropriate strains *Bacillus megaterium* AFI1 and *Paenibacillus nicotinae* AFI2.
